# {6,6′-Dimethoxy-2,2′-[cyclo­hexane-1,2-diylbis(nitrilo­methyl­idyne)]diphenolato-κ^4^ *O* ^1^,*N*,*N*′,*O* ^1′^}iron(II) monohydrate

**DOI:** 10.1107/S1600536809022235

**Published:** 2009-06-27

**Authors:** Peng-Fei Yan, Yan Bao, Hong-Feng Li, Guang-Ming Li

**Affiliations:** a School of Chemistry and Materials Science, Heilongjiang University, Harbin 150080, People’s Republic of China

## Abstract

In the title complex, [Fe(C_22_H_24_N_2_O_4_)]·H_2_O, the Fe^II^ center is four-coordinated by two O and two N atoms from 2,2′-[6,6′-dimethoxy­cyclo­hexane-1,2-diylbis(nitrilo­methyl­idyne)]diphen­olate (*L*) ligands in a distorted square-planar geometry. Uncoordinated water and Fe*L* mol­ecules are paired *via* inter­molecular water–meth­oxy O—H⋯O hydrogen bonds.

## Related literature

For a manganese complex of a similar Schiff base ligand, see: Watkinson *et al.* (1999 ▶). For the isotypic Co^II^ compound, see: Bao *et al.* (2009 ▶).
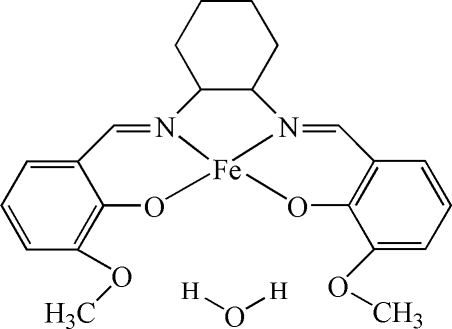


## Experimental

### 

#### Crystal data

[Fe(C_22_H_24_N_2_O_4_)]·H_2_O
*M* *_r_* = 454.30
Monoclinic, 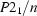

*a* = 11.243 (5) Å
*b* = 10.617 (3) Å
*c* = 17.863 (7) Å
β = 107.042 (14)°
*V* = 2038.5 (13) Å^3^
*Z* = 4
Mo *K*α radiation
μ = 0.78 mm^−1^
*T* = 291 K
0.22 × 0.21 × 0.18 mm

#### Data collection

Rigaku R-AXIS RAPID diffractometer
Absorption correction: multi-scan (*ABSCOR*; Higashi, 1995 ▶) *T*_min_ = 0.846, *T*_max_ = 0.873
18680 measured reflections
4584 independent reflections
3446 reflections with *I* > 2σ(*I*)
*R*_int_ = 0.054

#### Refinement

*R*[*F* ^2^ > 2σ(*F* ^2^)] = 0.042
*wR*(*F* ^2^) = 0.107
*S* = 1.03
4584 reflections
273 parameters
H-atom parameters constrained
Δρ_max_ = 0.30 e Å^−3^
Δρ_min_ = −0.35 e Å^−3^

### 

Data collection: *RAPID-AUTO* (Rigaku, 1998 ▶); cell refinement: *RAPID-AUTO*; data reduction: *CrystalStructure* (Rigaku/MSC, 2002 ▶); program(s) used to solve structure: *SHELXS97* (Sheldrick, 2008 ▶); program(s) used to refine structure: *SHELXL97* (Sheldrick, 2008 ▶); molecular graphics: *SHELXTL* (Sheldrick, 2008 ▶); software used to prepare material for publication: *SHELXL97*.

## Supplementary Material

Crystal structure: contains datablocks global, I. DOI: 10.1107/S1600536809022235/cv2567sup1.cif

Structure factors: contains datablocks I. DOI: 10.1107/S1600536809022235/cv2567Isup2.hkl

Additional supplementary materials:  crystallographic information; 3D view; checkCIF report

## Figures and Tables

**Table 1:** Hydrogen-bond geometry (Å, °)

*D*—H⋯*A*	*D*—H	H⋯*A*	*D*⋯*A*	*D*—H⋯*A*
O5—H2⋯O2	0.85	2.15	2.915 (4)	149
O5—H1⋯O4	0.85	2.06	2.898 (4)	169

**Fig. 1.**
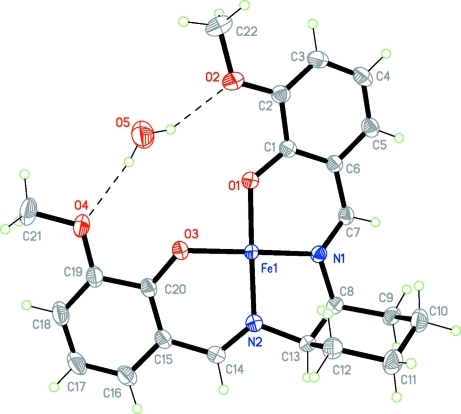
View of the title compound showing the atomic numbering and 30% probability displacement ellipsoids. Dashed lines indicate the hydrogen-bonding interactions.

## supplementary crystallographic information

### Comment

In the title compound (Fig. 1), the Fe^II^ ion is four-coordinated by the tetradentate Schiff base ligand in a square planar environment in a manner observed earlier for a manganese complex (Watkinson *et al.*, 1999). Uncoordinated water molecule is paired with the main molecule by the O—H···O hydrogen bonds (Table 1, Fig. 1).

### Experimental

The title complex was obtained by the treatment of anhydrous ferrous chloride with the Schiff base in methanol/acetone (2:3). The yellow clear mixture turned to black precipitation immediately, stirred for 4 h; diethyl ether was allowed to diffuse slowly into the solution of the filtrate. Black single crystals were obtained after several days. Analysis calculated for C_22_H_26_Fe_N_2_O_5: C, 58.16; H, 5.77; N, 6.17; Fe, 12.29; found: C, 57.56; H, 5.23; N, 6.77; F, 12.79%.

### Refinement

H atoms bound to C atoms were placed in calculated positions and treated as riding on their parent atoms, with C—H = 0.93 Å (aromatic C), C—H = 0.97 Å (methylene C), C—H = 0.98 Å (methine C), and with *U*_iso_(H) = 1.2*U*_eq_(C) or C—H = 0.96 Å (methly C) and with *U*_iso_(H) = 1.5*U*_eq_(C). Water H atoms were initially located in a difference Fourier map, but they were treated as riding on their parent atoms with O—H = 0.85 Å and with with *U*_iso_(H) = 1.5*U*_eq_(O).

### Crystal data

**Table:**

[Fe(C_22_H_24_N_2_O_4_)]·H_2_O	*F*(000) = 952
*M**_r_* = 454.30	*D*_x_ = 1.480 Mg m^−^^3^
Monoclinic, *P*2_1_/*n*	Mo *K*α radiation, λ = 0.71073 Å
Hall symbol: -P 2yn	Cell parameters from 13436 reflections
*a* = 11.243 (5) Å	θ = 3.1–27.5°
*b* = 10.617 (3) Å	µ = 0.78 mm^−^^1^
*c* = 17.863 (7) Å	*T* = 291 K
β = 107.042 (14)°	Block, black
*V* = 2038.5 (13) Å^3^	0.22 × 0.21 × 0.18 mm
*Z* = 4	

### Data collection

**Table:**

Rigaku R-AXIS RAPID diffractometer	4584 independent reflections
Radiation source: fine-focus sealed tube	3446 reflections with *I* > 2σ(*I*)
graphite	*R*_int_ = 0.054
ω scans	θ_max_ = 27.5°, θ_min_ = 3.1°
Absorption correction: multi-scan (*ABSCOR*; Higashi, 1995)	*h* = −14→14
*T*_min_ = 0.846, *T*_max_ = 0.873	*k* = −13→13
18680 measured reflections	*l* = −23→23

### Refinement

**Table:**

Refinement on *F*^2^	Primary atom site location: structure-invariant direct methods
Least-squares matrix: full	Secondary atom site location: difference Fourier map
*R*[*F*^2^ > 2σ(*F*^2^)] = 0.042	Hydrogen site location: inferred from neighbouring sites
*wR*(*F*^2^) = 0.107	H-atom parameters constrained
*S* = 1.03	*w* = 1/[σ^2^(*F*_o_^2^) + (0.0436*P*)^2^ + 1.092*P*] where *P* = (*F*_o_^2^ + 2*F*_c_^2^)/3
4584 reflections	(Δ/σ)_max_ = 0.001
273 parameters	Δρ_max_ = 0.30 e Å^−^^3^
0 restraints	Δρ_min_ = −0.35 e Å^−^^3^

### Special details

**Table:**

Geometry. All e.s.d.'s (except the e.s.d. in the dihedral angle between two l.s. planes) are estimated using the full covariance matrix. The cell e.s.d.'s are taken into account individually in the estimation of e.s.d.'s in distances, angles and torsion angles; correlations between e.s.d.'s in cell parameters are only used when they are defined by crystal symmetry. An approximate (isotropic) treatment of cell e.s.d.'s is used for estimating e.s.d.'s involving l.s. planes.
Refinement. Refinement of *F*^2^ against ALL reflections. The weighted *R*-factor *wR* and goodness of fit *S* are based on *F*^2^, conventional *R*-factors *R* are based on *F*, with *F* set to zero for negative *F*^2^. The threshold expression of *F*^2^ > σ(*F*^2^) is used only for calculating *R*-factors(gt) *etc*. and is not relevant to the choice of reflections for refinement. *R*-factors based on *F*^2^ are statistically about twice as large as those based on *F*, and *R*- factors based on ALL data will be even larger.

### Fractional atomic coordinates and isotropic or equivalent isotropic displacement parameters (Å^2^)

**Table:**

	*x*	*y*	*z*	*U*_iso_*/*U*_eq_	
Fe1	0.65626 (3)	0.46981 (3)	0.162776 (18)	0.02766 (11)	
N1	0.7304 (2)	0.52432 (19)	0.08782 (12)	0.0356 (5)	
O1	0.55829 (18)	0.34802 (16)	0.09933 (10)	0.0414 (4)	
O3	0.57113 (19)	0.43064 (17)	0.23484 (11)	0.0447 (5)	
N2	0.7650 (2)	0.5782 (2)	0.23003 (12)	0.0397 (5)	
C7	0.6991 (3)	0.4904 (2)	0.01541 (15)	0.0385 (6)	
H7	0.7339	0.5350	−0.0178	0.046*	
C1	0.5517 (2)	0.3230 (2)	0.02601 (15)	0.0370 (6)	
C2	0.4762 (3)	0.2205 (3)	−0.01171 (17)	0.0439 (6)	
C20	0.5684 (3)	0.5024 (2)	0.29420 (15)	0.0401 (6)	
O4	0.3917 (2)	0.3866 (2)	0.29798 (13)	0.0597 (6)	
C19	0.4729 (3)	0.4826 (3)	0.33048 (17)	0.0481 (7)	
O2	0.4206 (2)	0.1564 (2)	0.03582 (13)	0.0636 (6)	
C8	0.8176 (3)	0.6325 (2)	0.11520 (15)	0.0394 (6)	
H8	0.7676	0.7097	0.1075	0.047*	
C14	0.7552 (3)	0.6262 (2)	0.29455 (15)	0.0414 (6)	
H14	0.8169	0.6814	0.3220	0.050*	
C6	0.6156 (2)	0.3903 (2)	−0.01837 (15)	0.0372 (6)	
C4	0.5227 (3)	0.2627 (3)	−0.13222 (17)	0.0517 (7)	
H4	0.5117	0.2439	−0.1847	0.062*	
C15	0.6547 (3)	0.5989 (3)	0.32603 (15)	0.0421 (6)	
C5	0.5980 (3)	0.3603 (3)	−0.09773 (16)	0.0464 (7)	
H5	0.6381	0.4075	−0.1269	0.056*	
C13	0.8726 (3)	0.6129 (2)	0.20281 (16)	0.0411 (6)	
H13	0.9091	0.6918	0.2277	0.049*	
C12	0.9700 (3)	0.5089 (3)	0.22201 (18)	0.0502 (7)	
H12A	0.9306	0.4287	0.2040	0.060*	
H12B	1.0067	0.5038	0.2783	0.060*	
C3	0.4622 (3)	0.1911 (3)	−0.08882 (17)	0.0495 (7)	
H3	0.4125	0.1234	−0.1121	0.059*	
C9	0.9182 (3)	0.6509 (3)	0.07480 (17)	0.0486 (7)	
H9A	0.8798	0.6520	0.0186	0.058*	
H9B	0.9578	0.7319	0.0899	0.058*	
C10	1.0165 (3)	0.5485 (3)	0.09490 (19)	0.0594 (8)	
H10A	1.0822	0.5690	0.0719	0.071*	
H10B	0.9797	0.4692	0.0727	0.071*	
C11	1.0719 (3)	0.5339 (4)	0.1833 (2)	0.0648 (9)	
H11A	1.1307	0.4646	0.1943	0.078*	
H11B	1.1165	0.6101	0.2049	0.078*	
C18	0.4656 (3)	0.5554 (3)	0.3928 (2)	0.0633 (9)	
H18	0.4018	0.5412	0.4152	0.076*	
C21	0.2942 (3)	0.3601 (4)	0.3316 (2)	0.0694 (10)	
H21A	0.3291	0.3303	0.3843	0.104*	
H21B	0.2405	0.2967	0.3012	0.104*	
H21C	0.2472	0.4354	0.3322	0.104*	
C17	0.5525 (4)	0.6499 (3)	0.4229 (2)	0.0674 (10)	
H17	0.5468	0.6982	0.4651	0.081*	
C16	0.6452 (3)	0.6712 (3)	0.39035 (18)	0.0551 (8)	
H16	0.7032	0.7342	0.4106	0.066*	
O5	0.3096 (3)	0.3010 (3)	0.13694 (18)	0.1061 (11)	
H1	0.3424	0.3299	0.1827	0.159*	
H2	0.3649	0.2594	0.1238	0.159*	
C22	0.3307 (3)	0.0621 (3)	0.0015 (2)	0.0666 (10)	
H22A	0.2609	0.1004	−0.0360	0.100*	
H22B	0.3032	0.0219	0.0416	0.100*	
H22C	0.3674	0.0005	−0.0243	0.100*	

### Atomic displacement parameters (Å^2^)

**Table:**

	*U*^11^	*U*^22^	*U*^33^	*U*^12^	*U*^13^	*U*^23^
Fe1	0.02801 (19)	0.03099 (18)	0.02628 (18)	−0.00228 (14)	0.01153 (13)	−0.00086 (13)
N1	0.0334 (12)	0.0387 (11)	0.0353 (11)	−0.0007 (9)	0.0108 (9)	0.0018 (9)
O1	0.0425 (11)	0.0466 (10)	0.0377 (10)	−0.0046 (8)	0.0157 (8)	−0.0025 (7)
O3	0.0515 (12)	0.0491 (10)	0.0391 (10)	−0.0053 (9)	0.0221 (9)	−0.0030 (8)
N2	0.0406 (13)	0.0432 (12)	0.0382 (12)	−0.0011 (10)	0.0163 (10)	−0.0013 (9)
C7	0.0384 (15)	0.0438 (14)	0.0351 (14)	0.0024 (11)	0.0136 (12)	0.0046 (10)
C1	0.0351 (15)	0.0369 (13)	0.0386 (14)	0.0047 (10)	0.0101 (12)	−0.0025 (10)
C2	0.0398 (16)	0.0464 (15)	0.0470 (16)	0.0015 (12)	0.0148 (13)	−0.0043 (12)
C20	0.0409 (16)	0.0498 (15)	0.0318 (13)	0.0055 (11)	0.0141 (12)	0.0041 (10)
O4	0.0513 (14)	0.0833 (15)	0.0531 (13)	−0.0119 (11)	0.0289 (11)	0.0000 (11)
C19	0.0444 (18)	0.0620 (18)	0.0408 (16)	0.0010 (14)	0.0169 (13)	0.0047 (13)
O2	0.0712 (16)	0.0618 (13)	0.0629 (14)	−0.0281 (11)	0.0277 (12)	−0.0130 (10)
C8	0.0363 (15)	0.0393 (14)	0.0432 (15)	−0.0015 (11)	0.0129 (12)	0.0038 (11)
C14	0.0414 (16)	0.0463 (15)	0.0359 (14)	0.0002 (12)	0.0101 (12)	−0.0035 (11)
C6	0.0342 (14)	0.0418 (14)	0.0349 (13)	0.0054 (11)	0.0086 (11)	−0.0004 (10)
C4	0.0539 (19)	0.0608 (18)	0.0390 (16)	0.0104 (14)	0.0112 (14)	−0.0096 (13)
C15	0.0470 (17)	0.0469 (15)	0.0340 (14)	0.0011 (12)	0.0143 (12)	−0.0002 (11)
C5	0.0456 (18)	0.0559 (17)	0.0383 (15)	0.0046 (13)	0.0134 (13)	−0.0003 (12)
C13	0.0387 (16)	0.0445 (14)	0.0417 (15)	−0.0055 (11)	0.0143 (12)	−0.0025 (11)
C12	0.0397 (17)	0.0653 (19)	0.0439 (16)	0.0075 (13)	0.0098 (13)	0.0074 (13)
C3	0.0473 (18)	0.0480 (16)	0.0498 (17)	0.0019 (13)	0.0086 (14)	−0.0111 (13)
C9	0.0451 (18)	0.0587 (18)	0.0440 (16)	−0.0118 (13)	0.0162 (14)	0.0028 (12)
C10	0.0436 (19)	0.085 (2)	0.056 (2)	0.0016 (16)	0.0244 (16)	0.0000 (16)
C11	0.0369 (18)	0.098 (3)	0.061 (2)	0.0095 (17)	0.0161 (15)	0.0101 (18)
C18	0.057 (2)	0.093 (3)	0.0510 (19)	0.0029 (18)	0.0322 (17)	−0.0024 (16)
C21	0.046 (2)	0.106 (3)	0.065 (2)	−0.0016 (19)	0.0290 (17)	0.0186 (19)
C17	0.077 (3)	0.084 (2)	0.051 (2)	−0.001 (2)	0.0337 (19)	−0.0176 (17)
C16	0.062 (2)	0.0628 (19)	0.0436 (17)	0.0018 (15)	0.0206 (15)	−0.0097 (13)
O5	0.080 (2)	0.167 (3)	0.080 (2)	−0.021 (2)	0.0366 (17)	−0.0236 (19)
C22	0.055 (2)	0.0543 (19)	0.085 (3)	−0.0180 (15)	0.0124 (19)	−0.0009 (16)

### Geometric parameters (Å, °)

**Table:**

Fe1—N2	1.844 (2)	C4—H4	0.9300
Fe1—O1	1.8541 (18)	C15—C16	1.412 (4)
Fe1—O3	1.8623 (19)	C5—H5	0.9300
Fe1—N1	1.864 (2)	C13—C12	1.523 (4)
N1—C7	1.288 (3)	C13—H13	0.9800
N1—C8	1.496 (3)	C12—C11	1.525 (4)
O1—C1	1.317 (3)	C12—H12A	0.9700
O3—C20	1.313 (3)	C12—H12B	0.9700
N2—C14	1.294 (3)	C3—H3	0.9300
N2—C13	1.476 (3)	C9—C10	1.517 (4)
C7—C6	1.429 (4)	C9—H9A	0.9700
C7—H7	0.9300	C9—H9B	0.9700
C1—C6	1.410 (4)	C10—C11	1.525 (5)
C1—C2	1.423 (4)	C10—H10A	0.9700
C2—O2	1.373 (3)	C10—H10B	0.9700
C2—C3	1.376 (4)	C11—H11A	0.9700
C20—C15	1.411 (4)	C11—H11B	0.9700
C20—C19	1.424 (4)	C18—C17	1.394 (5)
O4—C19	1.378 (4)	C18—H18	0.9300
O4—C21	1.425 (4)	C21—H21A	0.9600
C19—C18	1.377 (4)	C21—H21B	0.9600
O2—C22	1.428 (4)	C21—H21C	0.9600
C8—C13	1.519 (4)	C17—C16	1.352 (5)
C8—C9	1.522 (4)	C17—H17	0.9300
C8—H8	0.9800	C16—H16	0.9300
C14—C15	1.431 (4)	O5—H1	0.8500
C14—H14	0.9300	O5—H2	0.8500
C6—C5	1.409 (4)	C22—H22A	0.9600
C4—C5	1.365 (4)	C22—H22B	0.9600
C4—C3	1.397 (4)	C22—H22C	0.9600
			
N2—Fe1—O1	174.23 (9)	C8—C13—C12	112.4 (2)
N2—Fe1—O3	93.78 (9)	N2—C13—H13	109.8
O1—Fe1—O3	86.17 (8)	C8—C13—H13	109.8
N2—Fe1—N1	85.62 (10)	C12—C13—H13	109.8
O1—Fe1—N1	95.05 (9)	C13—C12—C11	111.0 (2)
O3—Fe1—N1	173.78 (9)	C13—C12—H12A	109.4
C7—N1—C8	120.2 (2)	C11—C12—H12A	109.4
C7—N1—Fe1	125.91 (19)	C13—C12—H12B	109.4
C8—N1—Fe1	113.05 (16)	C11—C12—H12B	109.4
C1—O1—Fe1	126.75 (17)	H12A—C12—H12B	108.0
C20—O3—Fe1	124.53 (17)	C2—C3—C4	120.0 (3)
C14—N2—C13	119.1 (2)	C2—C3—H3	120.0
C14—N2—Fe1	127.8 (2)	C4—C3—H3	120.0
C13—N2—Fe1	113.07 (16)	C10—C9—C8	112.8 (2)
N1—C7—C6	125.7 (2)	C10—C9—H9A	109.0
N1—C7—H7	117.1	C8—C9—H9A	109.0
C6—C7—H7	117.1	C10—C9—H9B	109.0
O1—C1—C6	124.6 (2)	C8—C9—H9B	109.0
O1—C1—C2	118.5 (2)	H9A—C9—H9B	107.8
C6—C1—C2	116.9 (2)	C9—C10—C11	111.5 (3)
O2—C2—C3	124.5 (3)	C9—C10—H10A	109.3
O2—C2—C1	113.8 (2)	C11—C10—H10A	109.3
C3—C2—C1	121.7 (3)	C9—C10—H10B	109.3
O3—C20—C15	124.8 (3)	C11—C10—H10B	109.3
O3—C20—C19	118.8 (3)	H10A—C10—H10B	108.0
C15—C20—C19	116.4 (2)	C12—C11—C10	110.7 (3)
C19—O4—C21	117.7 (3)	C12—C11—H11A	109.5
C18—C19—O4	124.5 (3)	C10—C11—H11A	109.5
C18—C19—C20	121.2 (3)	C12—C11—H11B	109.5
O4—C19—C20	114.3 (2)	C10—C11—H11B	109.5
C2—O2—C22	118.4 (3)	H11A—C11—H11B	108.1
N1—C8—C13	105.1 (2)	C19—C18—C17	120.9 (3)
N1—C8—C9	116.7 (2)	C19—C18—H18	119.6
C13—C8—C9	111.8 (2)	C17—C18—H18	119.6
N1—C8—H8	107.6	O4—C21—H21A	109.5
C13—C8—H8	107.6	O4—C21—H21B	109.5
C9—C8—H8	107.6	H21A—C21—H21B	109.5
N2—C14—C15	123.6 (3)	O4—C21—H21C	109.5
N2—C14—H14	118.2	H21A—C21—H21C	109.5
C15—C14—H14	118.2	H21B—C21—H21C	109.5
C5—C6—C1	120.4 (2)	C16—C17—C18	119.8 (3)
C5—C6—C7	118.3 (2)	C16—C17—H17	120.1
C1—C6—C7	121.3 (2)	C18—C17—H17	120.1
C5—C4—C3	120.1 (3)	C17—C16—C15	120.7 (3)
C5—C4—H4	120.0	C17—C16—H16	119.6
C3—C4—H4	120.0	C15—C16—H16	119.6
C20—C15—C16	121.0 (3)	H1—O5—H2	107.8
C20—C15—C14	121.1 (2)	O2—C22—H22A	109.5
C16—C15—C14	117.8 (3)	O2—C22—H22B	109.5
C4—C5—C6	120.8 (3)	H22A—C22—H22B	109.5
C4—C5—H5	119.6	O2—C22—H22C	109.5
C6—C5—H5	119.6	H22A—C22—H22C	109.5
N2—C13—C8	104.4 (2)	H22B—C22—H22C	109.5
N2—C13—C12	110.5 (2)		

### Hydrogen-bond geometry (Å, °)

**Table:**

*D*—H···*A*	*D*—H	H···*A*	*D*···*A*	*D*—H···*A*
O5—H2···O1	0.85	2.52	3.102 (4)	126
O5—H2···O2	0.85	2.15	2.915 (4)	149
O5—H1···O3	0.85	2.69	3.254 (4)	125
O5—H1···O4	0.85	2.06	2.898 (4)	169
